# Editorial: Reviews in parasite & host

**DOI:** 10.3389/fcimb.2024.1391289

**Published:** 2024-04-03

**Authors:** Ummer R. Zargar, Viola Introini, João P. Assolini, Martina Paoletta

**Affiliations:** ^1^ Department of Zoology, Govt Degree College (GDC) Dooru, Jammu and Kashmir, Anantnag, India; ^2^ Department of Higher Education, Jammu and Kashmir, Anantnag, India; ^3^ European Molecular Biology Laboratory Barcelona, Barcelona, Spain; ^4^ Alto Vale do Rio do Peixe University, Caçador, Brazil; ^5^ Instituto Nacional de Tecnología Agropecuaria, Buenos Aires, Argentina

**Keywords:** parasite-host interactions, therapeutic targets, anti-cancer, parasitic diseases, protozoan

The interaction between hosts and parasites has fascinated researchers for centuries. Recent advances in molecular biology, genetics, bioinformatics, computational and AI-based technologies have helped to shed light on this complex relationship. However, the emergence of new infectious diseases such as COVID-19 has further highlighted the need to understand better the mechanisms at the core of pathogen-host interaction. It is important to note that many parasites such as protozoans (e.g. *Plasmodium*, *Schistosomes*, *Leishmania*, *Trypanosoma*, and *Entamoeba*), platyhelminths (e.g. various trematodes, cestodes), and Aschelminths (e.g. filarial worms, hookworms, Ascaris, whipworms) pose a significant threat to human and animal health worldwide. Despite being a prominent research area, it is also a neglected one, where limited funding holds the elimination of these infection diseases back.

The primary objective of this Research Topic, entitled **“** *Reviews in parasite & host*,” is to recapitulate the recent advancements in parasite-host interactions. The objective is to provide updated information on the current progress, discuss current and pressing research gaps, and clarify frontier areas requiring scholarly exploration (**Figure 1**). Additionally, this contribution will serve as a platform for budding parasitologists to acquaint themselves with the current state of parasite and host research. Furthermore, the ideas presented in this Research topic will unlock the prospects of parasitology and encourage young parasitologists to engage in advanced research to help combat various parasitic diseases. By providing the necessary information and insights into the current landscape of parasite-host interactions, this contribution will enable researchers to focus their efforts in the most efficient manner possible. This, in turn, will accelerate the research and progress towards developing effective treatments for parasitic diseases.

**Figure 1:**
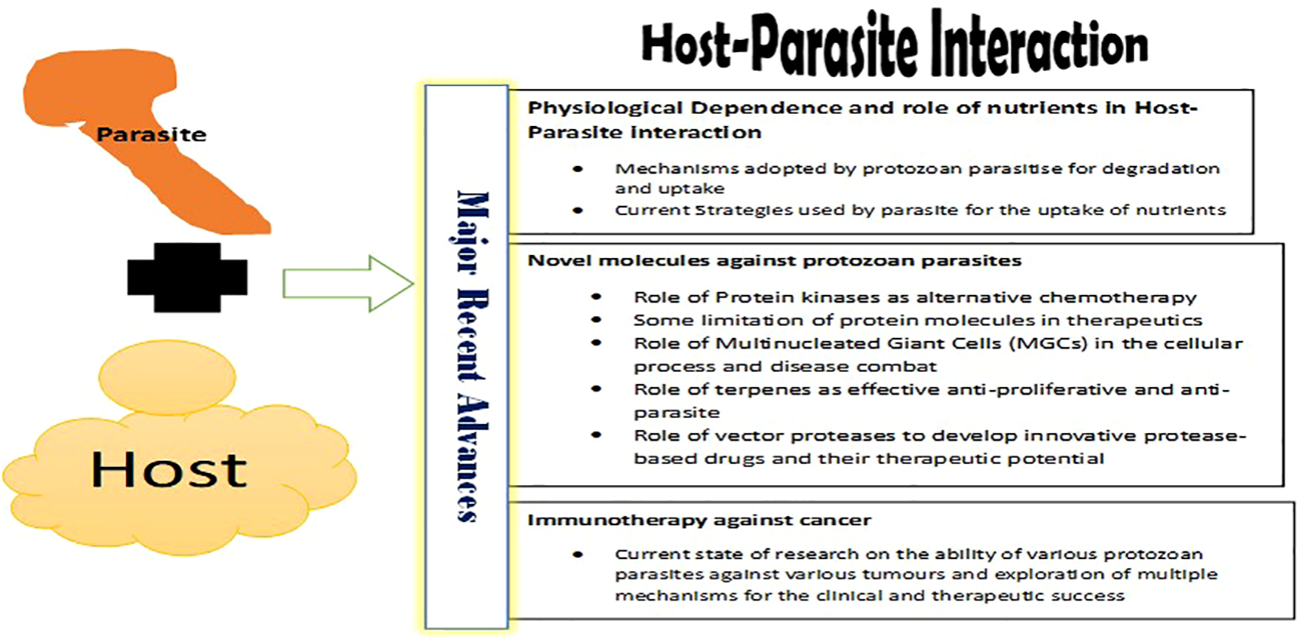
Current Advances in Host-Parasite Interaction.

The seven Original Research articles in this Research Topic deliberate upon three major themes related to host-parasitic interaction?

- Physiological dependence and host-parasitic interaction?
- What are therapeutic potential various molecules such as Protein Kinases (PKs) against protozoan parasitic diseases?
- What is the role of protozoan parasites in immunotherapy, especially anti-cancer therapy?

In recent years, there has been a significant amount of discussion surrounding the relationship between parasites and their hosts. Specifically, research has focused on the physiological dependence of parasites on their host’s ecological environment, impacting the parasite’s virulence and ability to survive within the host. Additionally, the potential for nutrient uptake by microbes has been found to play a role in the virulence factors and molecular mimicry exhibited by parasites. In their contribution to this Research Topic, Reyes-López et al. delves into the physiological aspects of hemoglobin uptake in protozoan parasites, shedding light on the current understanding of protozoan physiology about parasitic adaptation. Various tactics are utilized by parasites to ensure their survival and success. Among these is the utilization of proteases to extract iron from heme, which is then carried to the iron pool via specialized transporters. Nevertheless, additional exploration is required in order to completely comprehend the workings of this process. The realm of inquiry into this subject presents considerable potential for thrilling future inquiries. The more challenging physiological aspect addressed in this Research Topic is the unique strategy adopted by the trophozoite stage of the protozoan parasite *Entamoeba* that enabled it to thrive and show its reproductive potential in a challenging host environment. Based on the current status of research on various cell lines, such as Multinucleated Giant Cells (MGCs), it is imperative that researchers start working on the mechanistic pathways of these cell lines and their role in various host-parasite scenarios. By understanding this perspective at the molecular level, there are ample chances that we will be able to unravel the role of MGCs on epidemiological aspects of protozoan parasites and role in phenotypic variations, which will further help the researchers to find genomic and genetic changes at the host-parasite interactions.

The study of parasite-host interaction is a crucial area of research that holds promise for the development of effective therapeutic interventions. Researchers have faced significant challenges in treating many parasites with drugs. To overcome this hurdle, researchers have begun investigating the internal mechanisms that could modulate this interaction without the use of drugs. In their contribution to this Research Topic, Rodrigues et al. focuses on the treatment of Leishmaniasis, a parasitic disease, with a range of terpenes and various mechanisms, with emphasis on the role of immunomodulation, oxidative stress, and induction of parasite cell death. The findings of this research are significant, as they contribute to the understanding of the complex interactions between parasites and their hosts, and provide insights into the development of effective therapeutic interventions. Researchers have conducted extensive investigations into several natural products that demonstrate remarkable potential for developing effective drugs against *Leishmania* in recent decades. However, research on the effect of new molecules against protozoan parasites still needs to be unraveled. Recent studies have emphasized the significant role of triterpenes in combatting the severity of infection levels of *Leishmania* by stimulating immunomodulatory effects that increase the level of Th1 and decrease the level of Th2. Given the current status of the therapeutic potential of several new molecules, including triterpenes, there is an emerging potential in understanding the host-parasite interaction of *Leishmania* and its host in the bone marrow microenvironment. Therefore, it is crucial to delve deeper into these areas to understand better the potential therapeutic benefits of these molecules and their impact on the host-parasite interaction.

In the domain of parasite therapeutics, researchers have been exploring the use of protein therapies and other therapeutics as a potential solution to combat the protozoan parasitic diseases. This emerging aspect has gained significant attention in the post-genomic era. The review article in this Research Topic, by de Araújo et al. discusses the biotechnological potential of proteases found in hematophagous vectors and their role at the host-parasite interface. Additionally, protein kinases (PKs) have been found to be a useful alternative in treating several human protozoan diseases (dos Santos et al.). Researchers believe that exploring new kinase inhibitors could help find different routes of parasite cells. By examining the specificities of parasite PKs and their role in regulating energy metabolism, effective alternatives to current therapies could be found, reducing the side effects of existing treatments. Hazra et al. research on the examination of MGCs in *Entamoeba* not only sheds light on cell fusion in diverse organisms but also paves the way for further research on related cellular mechanisms and their implications for human health and disease. Furthermore, the research contribution of Veras et al. unraveled the immunological and hematological role of bone marrow in visceral leishmaniasis that could help to understand innovative therapeutic and diagnostic approaches in future.

Lastly, a novel interest is sparkling in the area focusing on the role of parasites in the immunotherapy against cancer. This aspect has been well described by Zheng et al. to demonstrate anti-tumor properties of protozoan parasites. Parasites were considered harmful to the host, nevertheless, recent studies suggest that protozoan parasites could be used to inhibit cancer occurrence and progression. Despite the potential anti-tumor effects of various protozoan parasites, there is a dearth of clinical trials involving human subjects. Moreover, the mechanisms underlying these effects remain poorly understood, and there is a general lack of interest among cancer researchers in exploring this avenue of treatment. These significant issues must be addressed before determining whether parasites, particularly protozoan parasites, could constitute a viable and universal form of cancer treatment.

In sum, the present research contributions under the title “*Reviews in parasite & host*” aims to provide a comprehensive overview of the recent advances in host-parasite interaction. This research contribution highlights some of the seminal findings, including the influence of the physiological micro-environment on the host-parasite interaction, the therapeutic potential of proteins and other biomolecules, and the latest developments in immunotherapy, particularly the use of protozoan parasites as anti-tumor agents. In view of the above, this research contribution is a valuable resource for scholars, scientists, and practitioners alike, as it offers insights into the complex mechanisms underlying host-parasite interactions and the potential therapeutic implications of these interactions. By providing a succinct and informative summary of the recent advances in this field, this article contributes to our understanding of the biology and pathology of parasitic infections and offers promising avenues for the development of novel therapeutics.

## Author contributions

UZ: Conceptualization, Writing – original draft, Writing – review & editing. VI: Writing – review & editing. JA: Writing – review & editing. MP: Writing – review & editing.

